# Photoactivation Transition State and Dynamical Response of the Orange Carotenoid Protein

**DOI:** 10.1021/acs.jpcb.5c05483

**Published:** 2025-12-04

**Authors:** Justin B. Rose, José A. Gascón, Damien I. Sheppard, Cheryl A. Kerfeld, Warren F. Beck

**Affiliations:** † Department of Chemistry, 3078 Michigan State University, 578 S. Shaw Lane, East Lansing, Michigan 48824, United States; ‡ Department of Chemistry, University of Connecticut, 55 N. Eagleville Road, Storrs, Connecticut 06269, United States; ¶ MSU−DOE Plant Research Laboratory, 3078 Michigan State University, 612 Wilson Road, East Lansing, Michigan 48824, United States; § Environmental Genomics and Systems Biology Division, 1438 Lawrence Berkeley National Laboratory, 1 Cyclotron Road, Berkeley, California 94720, United States; ∥ Molecular Biophysics and Integrated Bioimaging Division, 1438 Lawrence Berkeley National Laboratory, 1 Cyclotron Road, Berkeley, California 94720, United States

## Abstract

The orange carotenoid protein (OCP) regulates light harvesting in cyanobacteria by acting as a photoreceptor in its resting form, OCP^O^, and by effecting the quenching of bilin excitons upon binding to the core of the phycobilisome in its photoactivated red form, OCP^R^. We show herein using fluorescence anisotropy measurements and the action spectra for the rate constants of the two light-driven steps in the mechanism that the photoactivation of the OCP from *Synechocystis* sp. PCC 6803 is triggered by excited-state motions of the canthaxanthin chromophore that yield a twisted and bent conformation. Well-tempered metadynamics simulations reveal that a bicycle-pedal configuration, due to twisting of the two adjacent C=C bonds at the C13–C14 and C15–C15*′* positions in the center of canthaxanthin’s π-conjugated isoprenoid backbone, can be accommodated by the binding site in the OCP, with the energy of a captured photon required to cross the local activation energy barriers from the dark equilibrium structure. The bicycle-pedal configuration breaks the conserved hydrogen-bonding interactions between the carbonyl substituent of the β-ionone end ring of canthaxanthin and the adjacent W288 and Y201 residues in the C-terminal domain. The action spectra are modulated by the vibronic excitation prepared by absorption transitions to the S_2_ state, indicating that the photoactivation reactions are triggered by the canthaxanthin chromophore well prior to vibrational equilibration. These findings show that an ultrafast structural response of the OCP protein to the excited-state motions of the canthaxanthin chromophore controls the photoactivation yield and the sensing of blue light.

## Graphical abstract

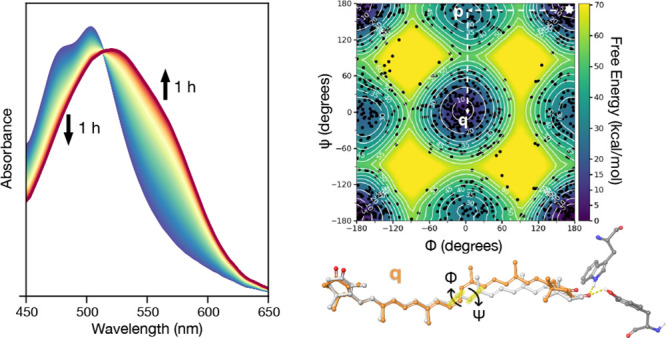

## Introduction

The orange carotenoid protein (OCP)^1–7^ performs photoprotective, nonphotochemical quenching functions in cyanobacteria.^8–11^ In *Synechocystis* sp. PCC 6803, the OCP is converted from its orange form, OCP^O^, to its red form, OCP^R^, via the sequence of two reversible steps shown in Figure 1, each driven forward by the absorption of a photon by a ketocarotenoid chromophore, canthaxanthin (CAN).^12^ The rate-limiting step in the overall mechanism is the photodissociation of the dark-stable OCP^O^ dimer to obtain a metastable monomeric intermediate, OCP^I^. The dark back reaction exhibits bimolecular kinetics, establishing that the OCP^O^ reactant is dimeric. The second step yields OCP^R^ by rotating away the C-terminal domain (CTD) and translocating the ketocarotenoid more than 12 Å into the N-terminal domain (NTD).^13^ An energetically favorable pathway involving a stepwise disengagement of the NTD–CTD interface has been elucidated computationally.^14^ This rearrangement results in a red-shifted and broadened absorption spectrum^12^ owing to the proximity of the CAN chromophore to several charged amino-acid side chains^11^ and to an enhancement of its conformational motions in the electronic ground state.^15^ These changes evidently enable OCP^R^ to quench bilin excitons in the phycobilisome upon assembling a dimer complex at the interface between the rods and the core, adjacent to the allophycocyanin segments ApcA and ApcB.^10,11^

**Figure 1:**
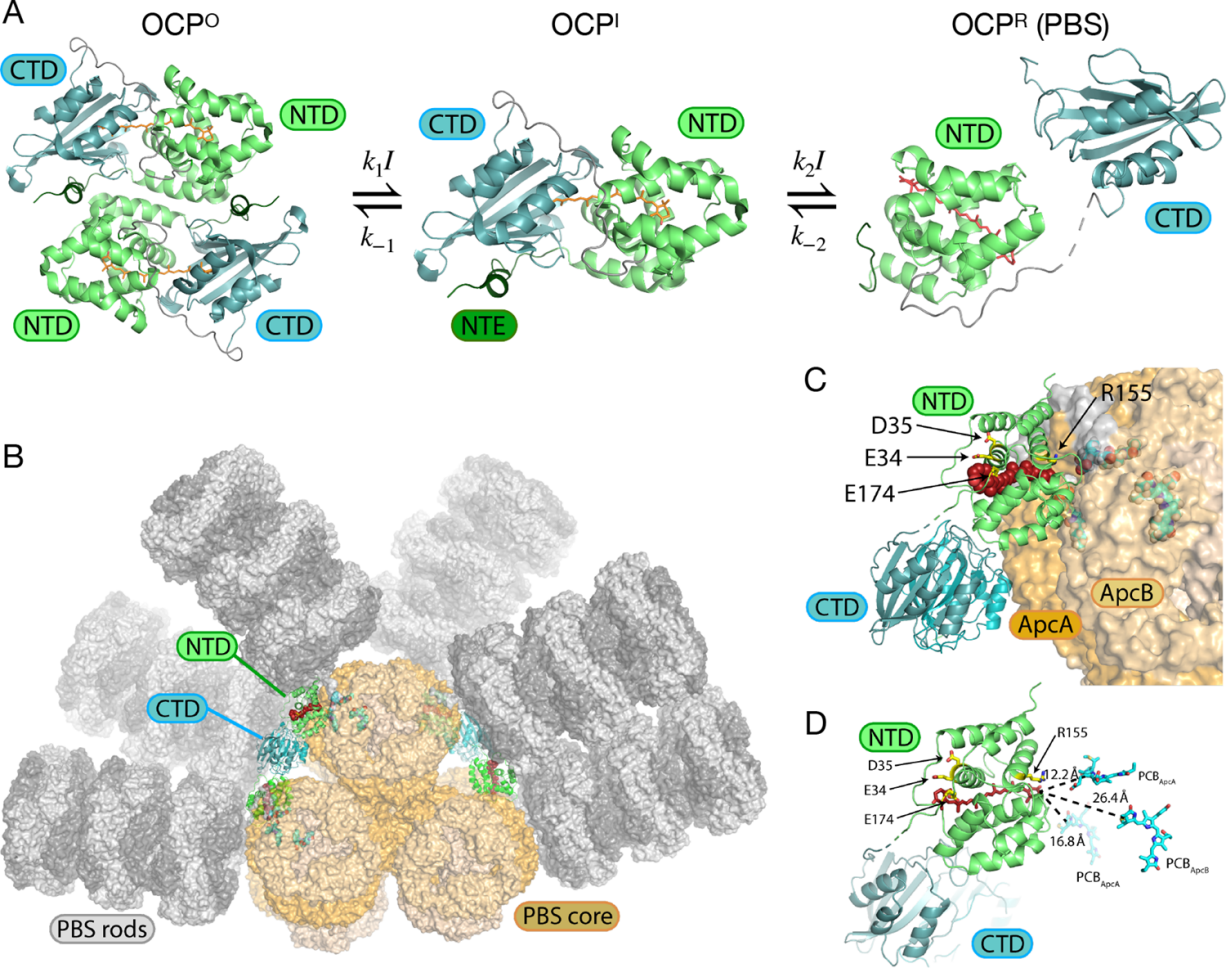
(A) Two-step photoactivation mechanism^12^ of OCP from *Synechocystis* sp. PCC 6803. The dimer OCP^O^ and monomer OCP^I^ are rendered from pdb id 4xb5,^13^ the former using crystallographic symmetry. OCP^R^(PBS), the phycobilisome (PBS)-bound form of OCP^R^, is rendered from pdb id 7sc9.^10^ The C-terminal (CTD) and N-terminal (NTD) domains are shown as blue and green ribbons, respectively, and the N-terminal extension (NTE) is shown as a dark green segment. The ketocarotenoid canthaxanthin (CAN) is drawn as an orange or red stick structure. The forward rate constants are expressed as *k*_1_ *I* and *k*_2_ *I* in terms of the light intensity *I*. Adapted with permission from ref 12. Copyright 2023, Royal Society of Chemistry. (B) Overview of the OCP^R^ dimer–phycobilisome complex of *Synechocystis* sp. PCC 6803,^10,11^ with a view of the phycobilisome core structure from pdb id 7SC9 and the individual rods merged from pdb id 7SCA.^10^ (C) OCP^R^ dimer bound to the allophycocyanin segments ApcA and ApcB, with CAN rendered in a red space-filling structure. (D) Expanded view of the OCP^R^–phycobilisome interaction, indicating several charged amino-acid side chains^11^ and the distances to three adjacent phycocyanobilin chromophores in the ApcA and ApcB segments. The structures shown in panels (C) and (D) are from pdb id 8TPJ.^11^ Panels (B–D) reproduced with permission from ref 15. Copyright 2024, AIP Publishing LLC.

How the ketocarotenoid in the OCP initiates the two light-driven reaction steps of the photoactivation mechanism has not been determined experimentally. Absorption of blue-green light prepares the S_2_ state of CAN, which decays nonradiatively in solution and in OCP preparations by first populating in <20 fs an intermediate, bridging state, S_*x*_,^16–19^ prior to decaying to the S_1_ state in ∼400 fs.^15,20^ At present, it is not known definitively if the CAN chromophore undergoes any excited-state motions during this relaxation pathway that would initiate formation of the OCP^I^ and OCP^R^ forms of OCP in the photoactivation mechanism. One of the key events in the second reaction, the breaking of the hydrogen-bonding interactions^2^ depicted in Figure 2 between the carbonyl on the β-ionone ring with the neighboring W288 and Y201 residues in the CTD, is proposed to follow population of the S_1_ state^21^ or from it the S* state^16^ lying at still lower energy.^22–24^ Translocation of the ketocarotenoid into the NTD requires a number of subsequent structural intermediates, which would follow the nonradiative recovery of CAN to the electronic ground state, S_0_.^22,25^ However, there have been no experimental studies yet of the dynamics of the two photoactivation steps, and the structural nature of the transition states has not been determined. The overall quantum yield for photoactivation is currently estimated as only ∼1%, from the yield of a nondecaying photoinduced absorption signal assigned to OCP^R^ in the ground electronic state, S_0_.^4^

**Figure 2:**
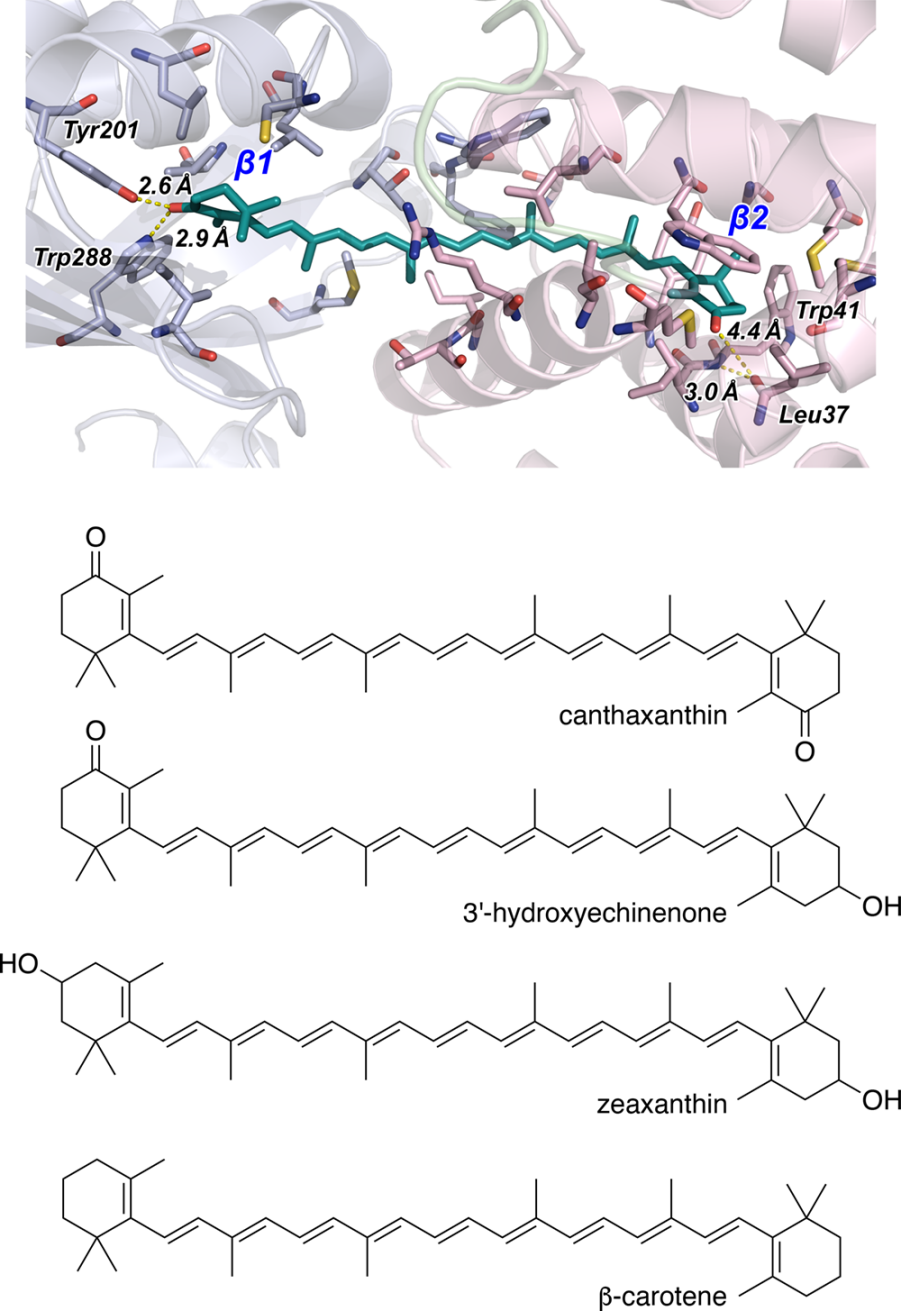
*Top panel:* binding site of canthaxanthin (CAN) in the structure of OCP^O^ from *Synechocystis* sp. PCC 6803,^13^ rendered from pdb id 4xb5. The C-terminal and N-terminal domains (CTD and NTD) of the OCP protein are rendered by gray and pink ribbons on the left and right, respectively. The CAN chromophore is rendered as a stick structure, and key interactions in the CTD and NTD are indicated with distance measurements. Hydrogen bonds are marked between the carbonyl substituent on the β-ionone ring β1 with Trp288 and Tyr201. Also, as noted previously,^26^ the carbonyl on the β2 ring clashes with the peptide carbonyl of Leu37, which then serves as a hydrogen-bond acceptor for the amide proton of Trp41. Note that protons are not shown in this diagram. Reproduced from ref 26. Copyright 2018, American Chemical Society. *Bottom panel:* Structures of canthaxanthin, 3*′*-hydroxyechinenone, zeaxanthin, and β-carotene.

In this contribution, we address these questions with new information from measurements of the fluorescence anisotropy of CAN and from the action spectra of the rate constants for the two light-driven reactions in the photoactivation mechanism. We show that light-induced conformational motions yielding a twisted and bent structure trigger the photoactivation reactions by driving coupled motions of the surrounding protein and solvent medium. This conclusion is based on the finding that the temperature dependence of the initial rate of photoactivation matches that of a decrease in the fluorescence anisotropy due to rotation of the emission transition dipole moment (TDM) as the CAN chromophore assumes the twisted structure. Molecular dynamics simulations show that a bicycle-pedal configuration of the ketocarotenoid, arising from twists at adjacent C=C bonds^27^ in the center of the isoprenoid backbone of CAN at the C13–C14 and C15–C15*′* positions, fits within the steric constraints of the binding site after local activation energy barriers from the ground-state structure are crossed. The action spectra reveal that the transition states of the two photoactivation reactions are prepared in a few hundred fs by the protein’s response to the light-driven motions of the ketocarotenoid, which initiates the subsequent reactions leading up to the reorganization of the protein structure on the μs–ms time scale.^22^ Taken together, these results show that the photoreceptor function and the sensing of blue light by the OCP is determined by the yields of the transition states for the two photoactivation reactions, which are controlled by an ultrafast structural response of the protein to the light-driven conformational motions of CAN.

## Experimental Methods

### Sample Preparation

OCP from *Synechocystis* sp. PCC 6803 was expressed with a C-terminal 6× His tag along with the plasmid for the biosynthesis for CAN, pAC-CANTHipi, in BL21­(DE3) competent *Escherichia coli* cells.^12,13,26^ One liter of culture was induced with isopropyl-β-D-thiogalactoside to a final concentration of 100 μM, allowed to grow 22 h at 25 °C, and then the cells were harvested by centrifugation at 9000*g*. The cells were suspended in a lysis buffer solution containing 50 mM tris­(hydroxymethyl)­aminomethane–NaOH, pH 8.0, and 200 mM NaCl, DNase at 5 mg/mL, and Sigmafast Protease Inhibitor, and then broken three times using a French pressure cell. The lysate was centrifuged at 41656*g* for 40 min and then purified by Ni-NTA affinity chromatography (HisTrap, GE Healthcare), producing a mixture of apo- and holoproteins. Ammonium sulfate precipitation of the mixture was performed with a final concentration of 1 M. The OCP–CAN holoprotein was isolated from the supernatant with hydrophobic interaction chromatography (HiTrap phenyl, GE Healthcare), bringing the ammonium sulfate concentration to 0 M, and then concentrated (Amicon 10 kDa centrifugal units, Sigma) before being stored frozen in a buffer solution containing 50 mM tris­(hydroxymethyl)­aminomethane–NaOH, pH 8.0, and 200 mM NaCl, with 10% (v/v) glycerol present as a cryoprotectant. Samples of the OCP–CAN holoprotein for fluorescence or photoactivation experiments were then handled as discussed previously.^12^ Frozen samples were thawed in total darkness and then diluted 30-fold with a 60/40 buffer/glycerol mixture to obtain an absorbance of 0.13 at 500 nm. The same sample conditions were used for the fluorescence spectra and for the measurements of the photoactivation rate constants for the action spectra. The glycerol is included here to obtain clear glasses for the fluorescence measurements at temperatures as low as 220 K. These samples were allowed to rest in a dark refrigerator for as long as a week prior to an experiment.

Canthaxanthin (Sigma/Aldrich 11775) was used as received. β-carotene (Sigma/Aldrich C9750–5G) was purified by HPLC as described previously^28,29^ by Amy LaFountain in the Frank laboratory at the University of Connecticut. The sample used here is from the same batch of β-carotene used in a previously published study of the fluorescence anisotropy of β-carotene^30^ and kept in dark storage in a –80° freezer for later use.

### Absorption and Fluorescence Spectroscopy

Fluorescence measurements were carried out with a home-built instrument, which was discussed in detail in previous work.^30,31^ The excitation light source was a broadband LED (Thorlabs M430L5, M455L4, M470L5, M490L4, or M505L4) and a double monochromator (Spectral Products AG1200-00500-303), which was used in the additive mode with 1200 gr/mm gratings to obtain a spectral bandpass of 2 nm. The M430L5 LED was used for excitation at 430 nm or nearby, but the M455L5, M470L5, M490L4, and M505L4 LEDs were used at longer excitation wavelengths. Fluorescence emission was detected by an Acton Research SP-150 spectrograph (300 gr/mm grating) and a Oxford Instruments Andor Newton 940-BV CCD detector. The monochromator and spectrograph were calibrated with an Ocean Optics HG-1 lamp; the absolute spectral response of the detection system was calibrated with an Ocean Optics HL-3 plus-CAL reference lamp.

The procedures we used to record fluorescence spectra were discussed in detail previously.^30,31^ The fluorescence anisotropy was measured using a pair of linearly polarized fluorescence spectra, recorded with the emission polarization at the magic angle, 54.7°, and then perpendicular to the excitation polarization. The equations required to calculate the anisotropy from the recorded spectra were derived previously^30^ and discussed in a subsequent review.^31^ This procedure obtains a better signal/noise ratio for the anisotropy spectrum because the saturation of the CCD detector by the highly polarized Raman scattering features during long exposures is avoided.^31^ The power of the excitation beam detected at the sample was 130 μW over the 430–450 nm range, 80 μW for 460–470 nm, and 30 μW for 480–500 nm, as determined by the choice of the LED placed prior to the excitation monochromator. The excitation wavenumber is marked on each of the plotted fluorescence spectra. Each set of spectra was obtained by integrating those from ten 150-s exposures of the CCD, and 6 cycles of the two polarizations were acquired, a total acquisition time of 2.5 h. The excitation polarizer’s rotation stage was set manually for the results reported here. The acquisition of data from the CCD was automated using LabVIEW (National Instruments) routines.

Samples were held in cryogenic cuvettes in helium exchange gas at temperatures over the 77–300-K range in a liquid nitrogen cooled cryostat (Oxford Instruments Optistat DN, with a Mercury iTC temperature controller). OCP samples were dark-adapted using the procedures described previously^12^ and then placed in the cryostat. CAN or β-carotene in 2-methyltetrahydrofuran (2-MTHF) was ramped down to 77 K over 1 h and then equilibrated there to obtain a clear glass. OCP samples were ramped down to 220 K and then equilibrated, again over 1 h. Temperature ramps up to a chosen temperature were followed by 10 min of equilibration prior to the recording of spectra. Absorption spectra were recorded with the sample in-place in the cryostat using a single beam, fiber-optic spectrophotometer consisting of a continuous white-light probe beam (Ocean Optics DH-2000) and a compact spectrograph/CCD detector (Ocean Optics USB4000), which was controlled by LabVIEW routines.

### Photoactivation Rate Measurements

Photoactivation rates and rate constants were determined with dark-adapted OCP preparations using the measurement conditions and protocols reported previously in detail.^12^ Absorption spectra of OCP for the photoactivation rate assays were measured with a single beam fiber-optic spectrophotometer, which consisted of a continuous white-light probe beam (Ocean Optics DH-2000) and a compact spectrograph/CCD detector (Ocean Optics Flame-T). A continuous excitation beam incident at 90° from the probe beam was obtained from a LED (430–530 nm, Thorlabs M series) or from a tungsten halogen lamp (550–620 nm, Thorlabs OSL2), which was collected by a fiber bundle (Thorlabs OSL2FB) and then passed through an interference bandpass filter (Thorlabs FB type, 10 nm bandpass) immediately prior to being passed through the OCP sample. The power of the white-light probe beam and of the excitation beam incident upon the sample were measured using a Newport Model 835 power meter equipped with a 1 cm silicon photodiode.

The photoactivation rate constants reported here as action spectra, the rate constant as a function of wavenumber of the excitation light, were determined with global models^32^ of the response of the absorption spectra as a function of the illumination time calculated with the CarpetView program (Light Conversion). As discussed previously, we applied a linear spectrokinetic model with three compartments corresponding to the dark-stable OCP^O^, the intermediate OCP^I^, and the fully photactivated product OCP^R^, with the rate constants *k*_1_ and *k*_2_ as indicated in Figure 1, to model the time response of the absorption spectrum. The OCP samples were mounted in a Peltier-type temperature controller for these experiments. The illumination power for the assays was adjusted with neutral density filters to obtain 250–300-μW across the range of wavelengths. The rates reported here have been corrected for the actinic illumination power used for a given measurement and for the rate of photoactivation due to the white-light probe beam alone. The procedures for these measurements and the optical conditions were as specified previously in detail.^12^

For the initial photoactivation rate measurements conducted over the 220–320-K temperature range, the same OCP samples used for fluorescence measurements in the Oxford cryostat were employed, and the cryostat was moved with the sample in place already at the chosen temperature to the photoactivation spectrometer. The initial rate of photoactivation was then determined from the integrated rate of change of the absorption difference spectrum, illuminated – dark-adapted. This type of assay was also discussed previously.^12^

### Computational Methods

Well-tempered metadynamics (WT-metaD) calculations^33^ were performed starting from the structure of OCP^O^ from *Synechocystis* sp. PCC 6803, pdb id 4XB5,^13^ to produce a path to create bicycle-pedal configurations of the CAN chromophore driven by internal rotations with respect to two central C=C bonds. The protein and the solvent environment as well as the relaxation protocol were prepared according to our previous studies of ketocarotenoid translocation and domain separation in OCP.^14,25^ The collective variables (CVs) were defined as the dihedral angles C14*′*–C15*′*–C15–C14 (CV1), and C15–C14–C13–C12 (CV2). A bicycle-pedal configuration at this site is favored by the electronic structure of the π-conjugated isoprenoid backbone of canthaxanthin, as discussed below, and it is consistent with the conclusions of our previous studies of the structural origin of the fluorescence anisotropy of β-carotene.^30^ The width of the Gaussian potential was *w* = 5.0, and the initial height was *h*_0_ = 0.1 kcal mol^–1^. The deposition time was Δ*t* = 0.1 ps, and the bias factor of WT-metaD (*k*_B_Δ*T*) was set to 20 kcal/mol.

## Results and Discussion

### Fluorescence Anisotropy of Canthaxanthin

The light-induced conformational motions of CAN in solution or when bound in the OCP can be detected using the fluorescence anisotropy,^34^ $r=(I_{∥}-I_{⊥})/(I_{∥}+2I_{⊥})=\frac{2}{5}((3\cos^{2}\;\theta -1)/2)$ , which determines the rotation angle θ between the absorption and emission TDMs usually using measurements of the linearly polarized fluorescence intensities, *I*_∥_ and *I*_⊥_. (As noted above, to obtain a better signal/noise ratio, we instead measure the magic-angle fluorescence spectrum with 54.7° polarization and then calculate the anisotropy using it and the perpendicularly polarized spectrum using the equations derived previously.^30,31^ ) Figure 3 shows measurements of the fluorescence anisotropy spectrum, *r* as a function of emission wavenumber, for CAN in 2-MTHF solvent at two temperatures. At 77 K, the anisotropy value near the peak of the fluorescence spectrum over the 18,000–21,500 cm^–1^ range is 0.36, which corresponds to a rotation of the emission TDM by ∼15°. This is an average value, excluding the interfering, highly polarized Raman scattering peaks from the solvent. An anisotropy value of 0.4 would mean that the absorption and emission TDMs are aligned, but a value of 0.36 is comparable to that observed for rigid molecules, not undergoing a large change of structure during the emission time scale. In contrast, the anisotropy value averaged over the same wavenumber range of the fluorescence spectrum observed at 293 K is 0.09, which gives a rotation of the emission TDM by ∼48°. The calculated TDM direction of a carotenoid is usually well aligned with the π-conjugated electron density along the isoprenoid backbone.^30,35,36^ These measurements reveal that CAN undergoes excited-state motions that twist and/or bend its π-conjugated isoprenoid backbone, causing the emission TDM to rotate, but the frozen solvent cavity around the chromophore apparently hinders these motions at cryogenic temperatures.

**Figure 3:**
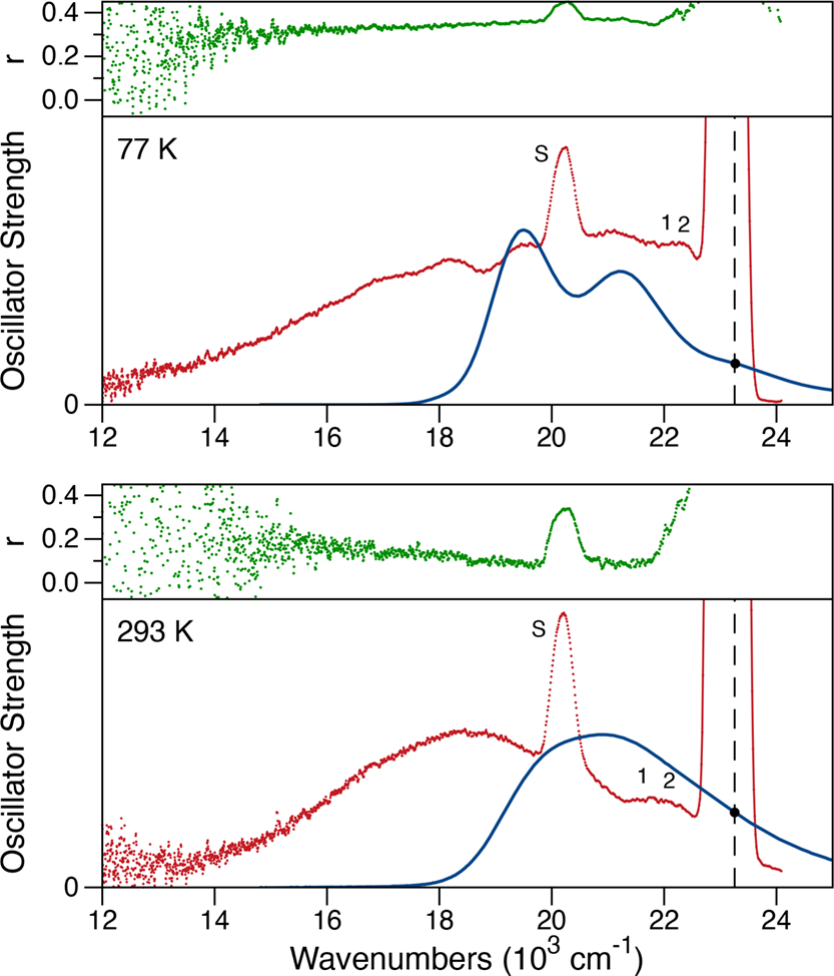
Absorption (blue), fluorescence emission (red), and fluorescence anisotropy *r* (green) spectra of canthaxanthin (CAN) in 2-methyltetrahydrofuran (2-MTHF) solvent at 77 and 293 K. The absorption and fluorescence spectra are plotted with arbitrary scaling as the oscillator strengths, ε­(ν)/ν and λ^2^ *F*(ν)/ν^3^, respectively. The fluorescence spectrum was recorded with the emission polarization at the magic angle, 54.7°, relative to the excitation polarization. The excitation source was tuned to 430 nm, as marked by the vertical dashed line at 23250 cm^–1^, which corresponds to excitation of the 0–2 vibronic transition. The positions of resonance Raman scattering peaks of canthaxanthin at 1517 and 1157 cm^–1^ are marked by the labels 1 and 2, respectively.^37^ The nonresonant Raman scattering peak from the 2-MTHF solvent at 2966 cm^–1^ is marked by the label S.^38^

To be detected via the fluorescence anisotropy, the time window for the excited-state motions of CAN is limited to the emission lifetimes of the S_2_ and S_*x*_ states. An assignment of the S_*x*_ state of carotenoids to a dark state with the 1^1^B_u_^–^ symmetry label, which would not be fluorescent, has been extensively discussed in the literature.^16–18,39^ However, as we noted previously,^40^ one can conclude that S_*x*_ is a fluorescent state on the basis of several experimental measurements. The fluorescence lifetime of β-carotene was determined to be ∼150 fs in femtosecond fluorescence upconversion experiments^41^ and in femtosecond Kerr-gate time-resolved fluorescence measurements.^42^ This lifetime is the same as that determined for the decay of S_*x*_ in femtosecond pump–probe^17^ and heterodyne transient grating^19^ experiments. In comparison, the lifetime of the S_2_ state of β-carotene is ultrashort, ∼10 fs.^17,19^ For CAN in toluene solution^20^ and in OCP^R^,^15^ a stimulated emission cross peak in the broadband two-dimensional electronic spectrum (2DES) is attributed to S_*x*_. The spectral bandwidth of the cross peak extends at least to the 14,000 cm^–1^ region. In comparison to the 14 fs lifetime of S_2_ for CAN in toluene, the 450 fs lifetime of S_*x*_ is long enough to allow the detection of out-of plane motions via the fluorescence anisotropy over the <1 ps time scale due to its spanning several periods of the midfrequency twisting and bending modes over the 200–1000 cm^–1^ range.^20^ Because the fluorescence anisotropy is normalized with respect to the total intensity of the fluorescence emission at a given detection wavenumber, *I*_∥_ + 2*I*_⊥_, it is insensitive to the kinetics of radiationless decay from the S_2_ state via the S_*x*_ intermediate, but a significant contribution from conformational motions in the S_1_ state during its 4.1 ps lifetime^20^ can essentially be ruled out. A weak fluorescence emission band due to S_1_ is observed in β-carotene at wavenumbers below ∼15,000 cm^–1^,^30,43^ but a comparable band is not resolved in the spectra from CAN.

The matching temperature thresholds for the rotation of the fluorescence emission TDM shown for CAN in Figure 4 and for β-carotene in Figure S3 coincide almost exactly with the glass-to-liquid dynamical transition of the 2-MTHF solvent at 142 K.^44,45^ This reveals that an increase in the specific volume and the activation of rotational motions of the surrounding solvent are required to allow the twisting motions to occur in the S_2_ and S_*x*_ states. The consensus thinking up to this point in the carotenoid literature has been that mostly planar structures are favored by long π-conjugation lengths,^16,46^ and recent calculations have not detected large-amplitude twisting motions of the isoprenoid backbone following optical preparation of the S_2_ state of CAN.^47^ The steric volume of a carotenoid would be expected to expand as it twists, but an accompanying induction of ICT character due to out-of-plane bending deformations^48,49^ would even more dramatically increase the strength of coupling of these motions to the rotations of polar groups in the surroundings. This idea is consistent with the trends exhibited by the fluorescence emission and anisotropy spectra of CAN, β-carotene, and zeaxanthin (ZEA), which are isostructural carotenoids other than for the substituents on the β-ionone rings, as depicted in Figure 2. The fluorescence anisotropy spectrum shown in Figure S4 for ZEA in 2-MTHF at 293 K determines an anisotropy value of ∼0.37, which suggests that the electron donating character of the alcohol substituents inhibits the twisting motions that accompany nonradiative decay from S_2_ to S_*x*_. The lower fluorescence anisotropy values of CAN compared to those of β-carotene correspondingly imply that the electron withdrawing character of the carbonyl substituents on the two β-ionone rings of CAN enhances the twisting motions by withdrawing π-electron density from the π-conjugated backbone.

**Figure 4:**
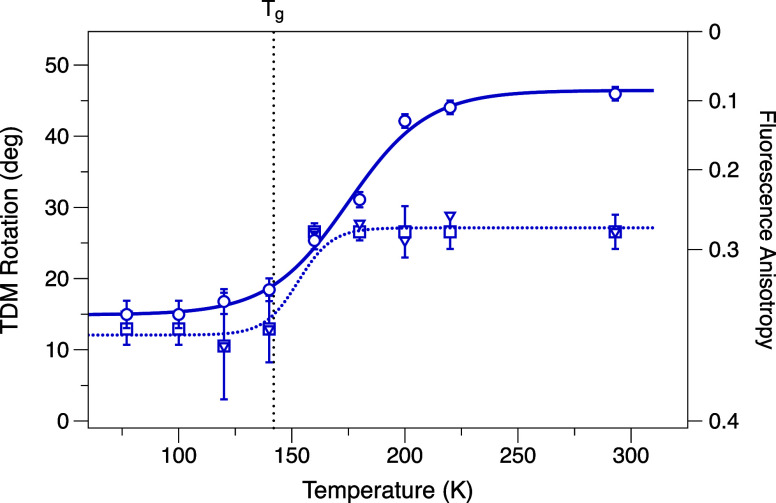
Rotation of the emission transition dipole moment (TDM) of CAN in 2-MTHF solution, as determined from measurements of the fluorescence anisotropy, with excitation of the (□) 0–0, (▽) 0–1, and (○) 0–2 absorption transitions. The excitation wavenumbers were scanned to track the wavenumbers of the vibronic transitions in the absorption spectrum as the sample temperature was changed, as shown in the Supporting Information in Figure S1. The fluorescence anisotropy spectrum for CAN at 293 K with excitation at the 0–0 vibronic transition is shown in Figure S2. The glass-to-liquid transition temperature for 2-MTHF, *T*_g_ = 142 K,^44^ is marked with a vertical dotted line.

### Fluorescence Anisotropy and Photoactivation of the OCP

Examples of the fluorescence emission and anisotropy spectra from CAN in dark-adapted OCP^O^ preparations at 220 and 293 K are shown in Figure 5. The temperature dependence of the fluorescence anisotropy values for OCP^O^ shown in Figure 6 indicates that excited-state conformational motions rotate the emission TDM of CAN even though the chromophore is bound inside the protein’s binding site. Almost exactly the same range of rotation is observed, but the temperature threshold occurs at ∼240 K, ∼100 K higher than observed in solution. This finding strongly suggests that the rotation of the emission TDM accompanies a local twisting distortion that minimizes the change in the steric volume of the isoprenoid backbone of CAN, such as the “bicycle-pedal”^27^ or “hula-twist” ^51,52^ configurations reached via the internal rotation of two adjacent C=C bonds. The ∼100-K increase in the temperature onset implies that the ketocarotenoid’s motions are strongly coupled to a dynamical transition of the surrounding protein, requiring thermal activation of long-range or collective motions and of the associated hydration shell.^53,54^

**Figure 5:**
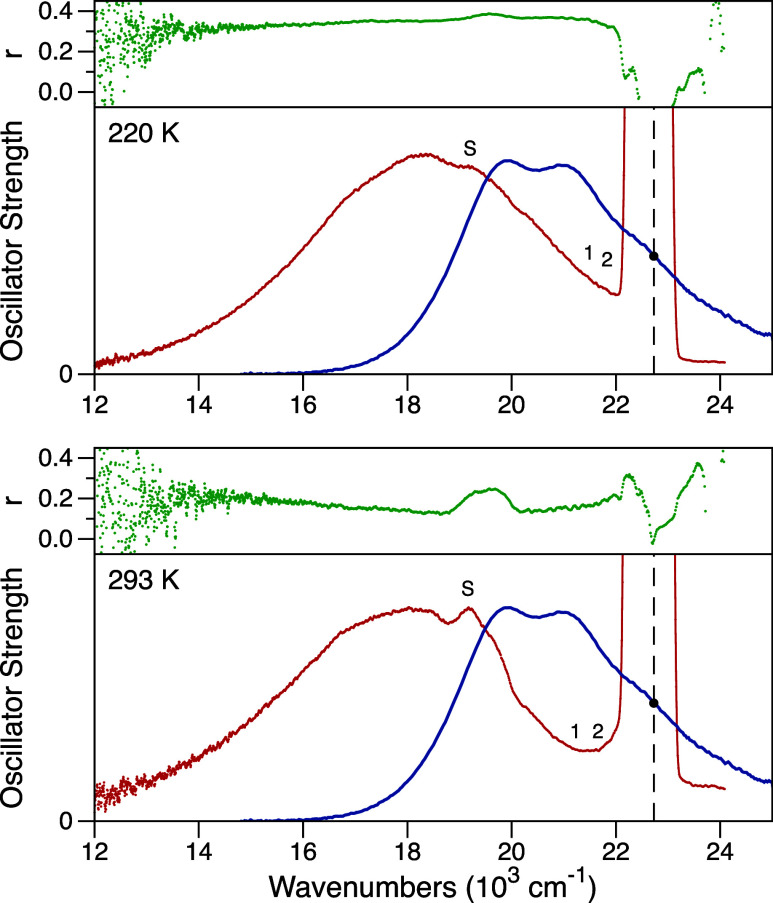
Absorption (blue), fluorescence emission (red), and fluorescence anisotropy *r* (green) spectra of resting, dark-adapted preparations of OCP^O^ at 220 and 293 K. The absorption and fluorescence spectra are plotted with arbitrary scaling as the oscillator strengths, ε­(ν)/ν and λ^2^ *F*(ν)/ν^3^, respectively. The fluorescence spectrum was recorded with the emission polarization at the magic angle, 54.7°, relative to the excitation polarization. The excitation source was tuned to 430 nm, as marked by the vertical dashed line at 23,250 cm^–1^, which corresponds to excitation of the 0–2 vibronic transition. The positions of resonance Raman peaks of canthaxanthin at 1517 and 1157 cm^–1^ are marked by the labels 1 and 2, respectively,^37^ and the nonresonant Raman scattering peak from water at 3500 cm^–1^ is marked by the label S.^50^

**Figure 6:**
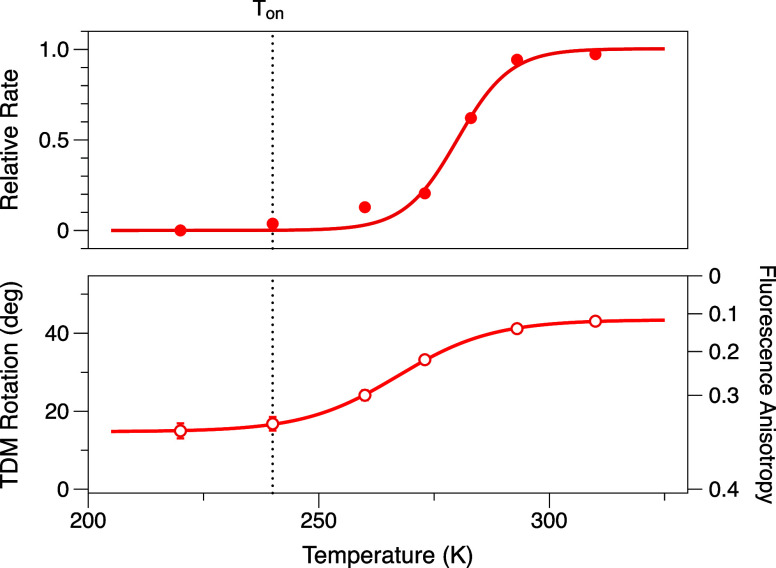
*Top panel*: initial rate of photoactivation of dark-adapted OCP^O^, relative to the rate at 310 K. *Bottom panel*: rotation of the emission transition dipole moment (TDM) for CAN in dark-adapted OCP^O^ as a function of temperature, as determined by fluorescence anisotropy measurements with excitation at the wavenumber of the 0–2 absorption transition. The same sample conditions were used for the photoactivation rate and fluorescence anisotropy measurements. A vertical dotted line at 240 K marks an estimate based on the two plots for the threshold temperature, *T*_on_.

The temperature dependence of the initial rate of photoactivation observed under the same measurement conditions, also shown in Figure 6, exhibits a sigmoidal temperature response matching that of the fluorescence anisotropy. This observation establishes that the excited-state twisting motions of CAN are required to advance along the reaction coordinate toward the OCP^R^ product of the photoactivation mechanism. (We can now preliminarily account for the lack of photoactivation activity in OCP preparations containing ZEA,^55^ given the finding here that ZEA does not undergo any twisting motions in 2-MTHF solution at 293 K.) Figure S5 shows an Arrhenius plot of the initial photoactivation rates. The activation energy is ∼8.5 kcal/mol, which apparently corresponds to the average energy required to activate the protein motions upon photoexcitation of CAN to the S_2_ state.

### Photoactivation Action Spectra

Additional information on the reaction dynamics of the two photoactivation steps of OCP can be gained from a study of their action spectra, the dependence of the rate constants *k*_1_ and *k*_2_ (Figure 1a) with respect to the wavenumber of the incident light. The rate constants were measured using the time evolution of the absorption spectrum under continuous illumination, as discussed previously.^12^ The response of the absorption spectrum was analyzed with a minimally determined global model^32^ with three spectrokinetic species,

$${\mathrm{OCP}}^{O}\overset{k_{1}}{\to }{\mathrm{OCP}}^{I}\overset{k_{2}}{\to }{\mathrm{OCP}}^{R}$$ 1

Only three species were detected by a principal component analysis using singular value decomposition (SVD), but as noted previously there may be additional components present in the sample at low concentrations.^12^ Figure S6 shows an example of these measurements, with excitation at 450 nm. The global model fits the absorption spectrum observed from the OCP ensemble at a certain illumination time as a linear combination of the basis spectra for the three spectrokinetic species, as scaled by the populations determined by the two time constants in the kinetic model, *k*_1_ = 1/τ_1_ and *k*_2_ = 1/τ_2_. The basis spectra are called evolution-associated difference spectra (EADS),^32^ which here represent the linear absorption spectrum of a particular spectrokinetic species. As plotted as absorption oscillator strength spectra, the EADS spectra measure the squares of the relative probability for absorption transitions as a function of the excitation wavenumber.^34,56^

Figure 7 compares the action spectra for *k*_1_ and *k*_2_ to the EADS spectra for the OCP^O^ and OCP^I^ reactants, respectively. Both of the action spectra are blue-shifted with respect to the EADS spectra, with that for *k*_2_ displaying an especially prominent lag in its low-frequency onset. Normalization of the action spectra with respect to the absorption oscillator strength, *k*_*i*_/*f*_abs_, reveals that the yields of both reactions increase markedly as the wavenumber of the incident illumination is tuned above the wavenumber of the 0–1 transition. This observation is mirrored by the increased rotation of the fluorescence TDM observed with excitation of the 0–2 transition compared to that with excitation of the 0–0 or 0–1 transitions, as shown in Figure 4.

**Figure 7:**
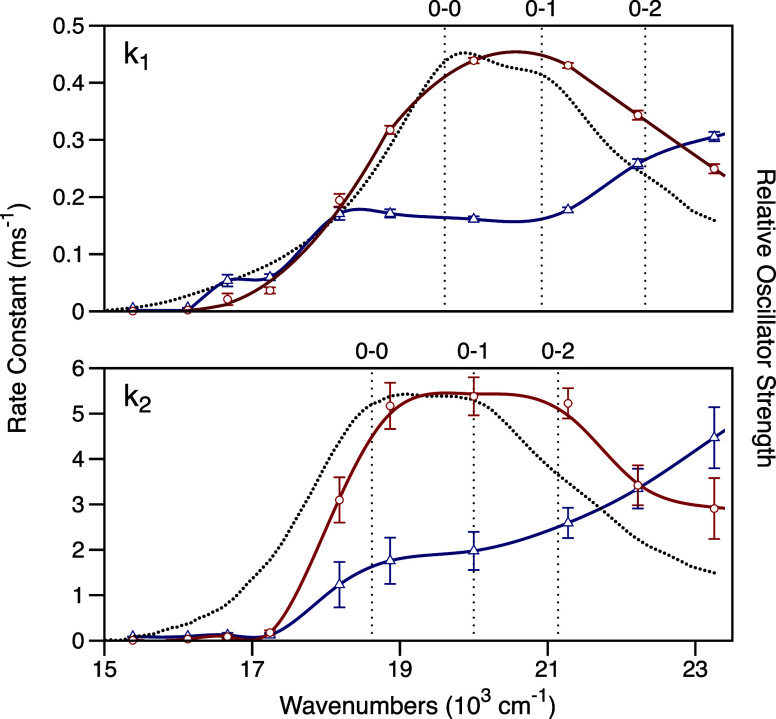
Action spectra (○, red curves) for the rate constants *k*_1_ and *k*_2_ of the two steps of the photoactivation mechanism of the OCP at 273 K, here plotted as a function of the wavenumber of the incident light. The absorption oscillator strength spectra *f*_abs_(ν) (black dotted curves) determined previously^12^ for the reactants OCP^O^ and OCP^I^, respectively, are plotted for comparison, with normalization to the peak maximum of the action spectrum. Vertical dotted lines are drawn to mark the wavenumbers of the 0–0, 0–1, and 0–2 vibronic transitions, as determined previously with numerical simulations with the multimode Brownian oscillator model.^12^ The ratios of the rate constants with the oscillator strength, *k*_*i*_/*f*_abs_, are then plotted (△, blue curves) with an arbitrary scaling of the ordinate. Error bars report the 95% confidence intervals from a set of replicate measurements.

The blue-shifted and vibronically modulated action spectra indicate that a potential-energy barrier must be passed over following optical preparation of the S_2_ state in order for the CAN chromophore to undergo out-of-plane distortions on the way to the S_1_ state.^30,40^ This idea was suggested previously in femtosecond pump–probe studies of β-carotene.^57^ However, the *k*_*i*_/*f*_abs_ ratio would be flat, independent of the wavenumber of the incident light, if the transition states of the photoactivation reactions were populated after vibrational cooling and thermal equilibration occurs during the lifetimes of the S_*x*_ and S_1_ states.^15,20^ This observation restricts the conformational motions of CAN certainly to the <400 fs time scale. Similar femtosecond time scales for the photochemistry of retinal in rhodopsin^58^ and bacteriorhodopsin^59^ have been directly observed with time-resolved spectroscopic methods, but the action spectra do not appear as strongly dependent on the excitation wavelength.^60^

As discussed previously using numerical simulations with the multimode Brownian oscillator model,^31,61,62^ the absorption oscillator strength spectrum of CAN is well described by a vibronic progression in the ω_1_ and ω_2_ C=C and C–C stretching coordinates of the isoprenoid backbone of CAN over the wavenumber range plotted for the action spectra. This argues against the suggestion that the increasing trend in the rate constants over the 17,000–23,000 cm^–1^ range arises from an excitation of an underlying weak absorption band in the blue part spectrum due perhaps to a twisted ground-state conformer. The values of the rate constants reflect the populations in the three spectrokinetic species used in the global models, and the EADS basis spectra that determine the populations have spectral origins in the green part of the spectrum, consistent with CAN chromophores with principally all-*trans* configurations. The absorption of ground-state carotenoids with *cis* configurations is observed over the 28,500–30,300 cm^–1^ (300–350 nm) range.^29,63^

### Molecular Dynamics Simulations

To gain additional insight on how the light-driven motions of CAN are coupled to the surrounding protein structure of the OCP, we performed a force-field-based metadynamics simulation^14,25,33^ to determine whether the Gibbs free-energy barriers adjacent to the structure of the equilibrium ground-state structure of CAN would permit the photochemical formation of bicycle-pedal configurations of the ketocarotenoid analogous to those proposed for the retinal chromophores in rhodopsin.^27^ Anisotropy responses comparable to those observed here for the fluorescence of CAN were previously observed in bacteriorhodopsin^64^ and rhodopsin^65^ in femtosecond fluorescence upconversion and pump–probe experiments, respectively.

A bicycle-pedal structure at the C9*′*–C8*′* and C7*′*–C6*′* positions near the β2 ring of CAN in the NTD (Figure 2) was observed in OCP crystals following illumination,^66^ but given the low quantum yield of photoactivation, this likely corresponds to the trapping of a side product in the major fraction of ketocarotenoid photoevents that do not proceed to form the photoactivated products. In fact, nonadiabatic molecular dynamics simulations of a *trans*-to-*cis* photoisomerization at the C8–C7 site near to the β1 ring, at the other end of the molecule, have been recently included in a discussion of the photoactivation mechanism.^47^ The bond-length alternation pattern in the S_2_ state,^28,67^ however, accounts for the finding that photoisomerization is strongly favored at the C15 position in β-carotene, at the center of the π-conjugated isoprenoid backbone.^52,67^

Figure 8a shows the free-energy surface of OCP^O^ in the ground electronic state, as determined by well-tempered metadynamics (WT-metaD) simulations^33^ with respect to the dihedral angles of two adjacent C=C bonds of CAN, at the C13–C14 and C15–C15*′* positions. The path shown in Figure 8a steers the ketocarotenoid to reach a bicycle-pedal configuration by performing successive out-of-plane 180° rotations of the two dihedral coordinates. The structures of the intermediate *p* and the bicycle-pedal product *q* are compared with the ground-state structure of CAN in Figure 8b. Conversion to the *p* and *q* intermediates will break or severely weaken the bifurcated hydrogen-bonding interaction^68^ between the carbonyl group on the β1 ring of CAN and the adjacent Y201 and W288 residues in the CTD because the effective length of the isoprenoid backbone is markedly shortened. The averages of these key bond lengths in the dark, *p*, and *q* states are listed in Tab. S1, and their distributions are compared in Figure S8. A representative snapshot from the WT-metaD simulations showing the distances and angles for the hydrogen bonds with Y201 and W288 is shown in Figure S9. The orientations in *p* and *q* of the carbonyl substituents on the two β-ionone rings, however, do not change much from that of the ground state, in line with observations of their transient absorption anisotropy in the mid-IR.^22^ The steric bulk of the isoprenoid backbone expands at the bicycle-pedal site, but the structures *p* and *q* do not clash significantly with the surrounding protein structure. Even for those residues in close contact with CAN, Figure 8c indicates that the average change in the root-mean-square fluctuation accompanying conversion from the dark structure to the *p* or *q* structure is about 0.2 Å, presenting a maximum of 1.25 Å for I151. Considering that CAN undergoes a substantial out-of-plane distortion during the proposed bicycle-pedal transition, these changes in fluctuations suggest that relatively small local displacements are imposed on the protein surroundings. A mutation of I151 to a less bulky side chain, however, might be expected to modulate the local packing and, in turn, have some impact on the dynamics of photoactivation.

**Figure 8:**
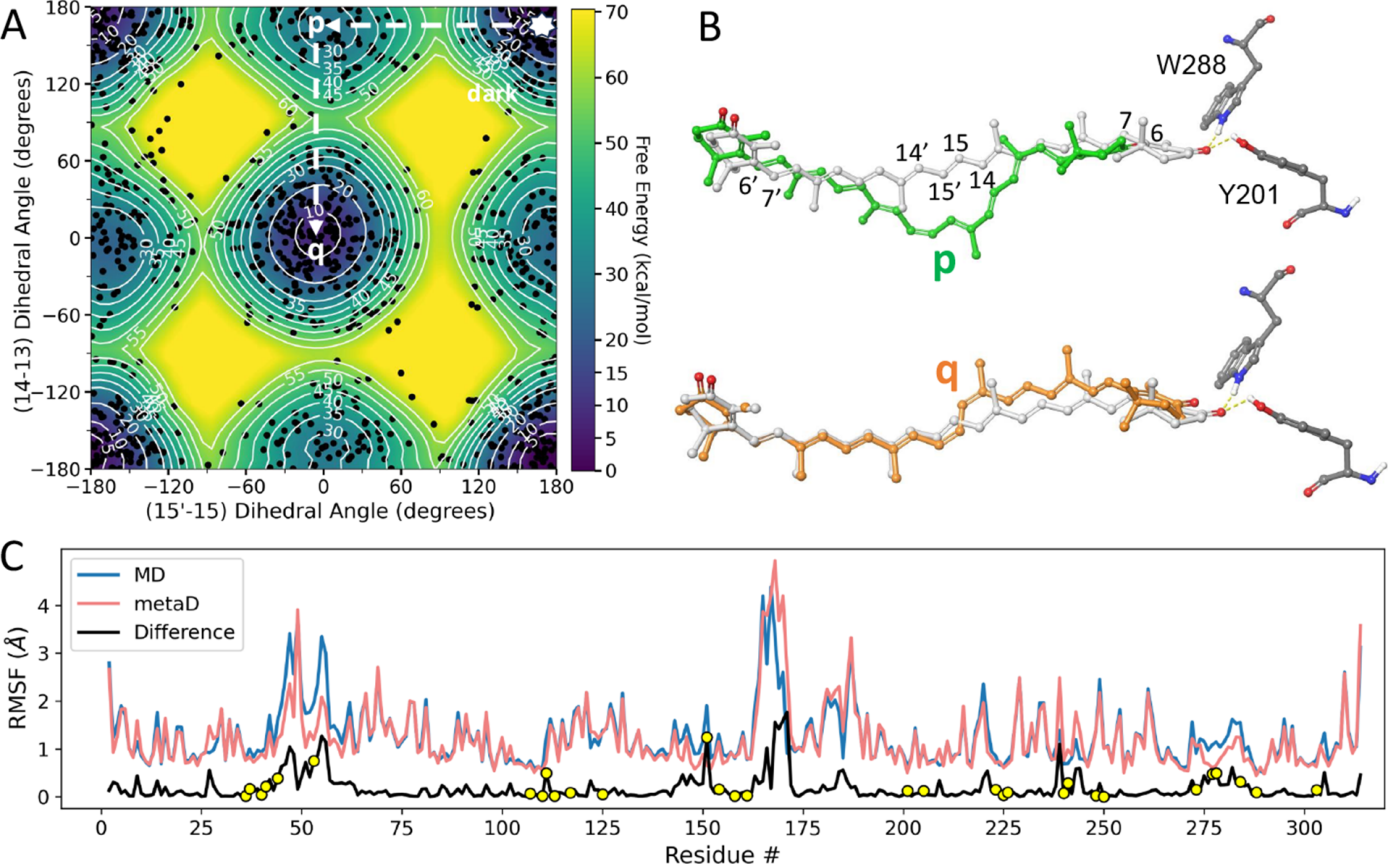
(A) Gibbs free-energy surface for CAN in OCP^O^ based on the pdb id 4XB5 structure^13^ from *Synechocystis* sp. PCC 6803. Dashed lines show the path from the global potential-energy minimum of the electronic ground state (∗) to reach a bicycle-pedal configuration due to sequential 180° rotations of the C=C bonds at (*p*) the C15–C15*′* and (*q*) the C13–C14 positions of CAN. With respect to the ground state, the energies of *p* and *q* are at ∼30 kcal/mol and ∼7 kcal/mol, respectively; for travel to *p* directly from the global minimum, the barrier is ∼55 kcal/mol, and the barrier from *p* to *q* is ∼20 kcal/mol. Figure S7 in the Supporting Information shows the corresponding potential surface for 2-MTHF solvent, which presents a ∼45 kcal/mol barrier between the dark state and *p* and between *p* and *q*. Black dots represent the distribution of snapshots collected during the metadynamics simulations. (B) Representative structures in the *p* (green) and *q* (orange) minima, each superimposed on the equilibrium ground-state structure (white) from the crystal structure. The hydrogen-bonding interactions (dashed bonds) between the carbonyl oxygen on the β-ionone end ring of CAN and the hydrogen-bonding donors on W288 and Y201 in the CTD are displaced especially in the *p* structure. (C) Root-mean-square fluctuations (RMSF) of the amino-acid side chains of OCP in the dark (blue trace) compared to those for the metadynamics simulations (pink trace). The difference between these two cases is shown by the black trace. Points highlighted with yellow circles correspond to the residues that are in van der Waals contact with CAN in OCP.

These WT-metaD calculations do not attempt to simulate the actual excited-state nonadiabatic dynamics that accompany the change in the π-conjugated structure of CAN along this pathway, but they provide accurate estimates of the strength of the local barriers that would restrict the motion of CAN in the dark and accordingly would mediate the mechanical coupling associated with the protein dynamical response. The free energy required to generate the *p* and *q* structures would be derived from the energy captured by the ketocarotenoid from an absorbed photon. The activation energy barrier from the ground-state minimum to that of *p* is ∼55 kcal/mol; the corresponding barrier in 2-MTHF solution is ∼45 kcal/mol (Figure S7). Despite the classical force-field approximations used to obtain these estimates, these barriers are comparable to the 40–50 kcal/mol barriers determined experimentally for the photoconversion of retinal in the visual and microbial rhodopsins.^69^

The rotation of the two C=C bonds to reach the bicycle-pedal structure *q* would be expected to accompany recovery to the electronic ground state, S_0_, after a nonadiabatic passage from the Franck–Condon S_2_ state structure through the conical intersections (CIs)^30,48,70–72^ with the S_*x*_ and S_1_ states. We propose that the fluorescence TDM is rotated by out-of-plane twisting dynamics of CAN near the CIs, where the three excited states are strongly mixed and there is considerable ICT character.^15^ These CIs may be similar to those determined for 2–4–6 octatriene.^73^ The theory proposed for π-conjugated molecules with conformationally dependent electronic structures, such as the flexible substituted benzene molecules and molecules with twisted intramolecular charge-transfer (TICT) states,^74^ accounts for large rotations of the fluorescence TDM in terms of state mixing and state reordering near CIs.

## Conclusions

The results discussed in this paper support the proposal that both steps of the photoactivation mechanism of OCP are triggered at temperatures above 240 K by light-induced twisting motions of the ketocarotenoid CAN following optical preparation of the S_2_ state, such as those producing a bicycle-pedal intermediate. The fluorescence anisotropy measurements return the average over the photoexcited ensemble, which indicates that the excited-state twisting motions of CAN can occur efficiently if the protein and solvent medium can undergo thermally activated motions of sufficient amplitude to accommodate the steric volume of the twisted, excited-state structure. However, given the low overall quantum yield of photoactivation, the entropy of activation^75^ of the two light-driven photoactivation reactions are both negative, Δ*S*_rxn_^‡^ < 0, meaning that the transition states of the two light-driven steps in the mechanism are relatively improbable. This means that although the surrounding protein and surrounding hydration shell are strongly coupled to the excited-state motions of CAN, as revealed by the sharp temperature onsets of the fluorescence anisotropy and of the initial photoactivation rate, only a small fraction of the photoevents results in a protein response that continues successfully along the photoactivation reaction coordinate.

The blue-shifted and vibronically modulated action spectra of the rate constants indicate that the productive transition states are populated after optical preparation of the S_2_ state but well prior to vibrational equilibration in the S_1_ state. These time scales are depicted in the cartoon shown in Figure 9, which is based on those determined for CAN in solution and for CAN in the OCP^R^ protein by 2DES measurements^15,20^ and on our previous discussion of the fluorescence anisotropy of β-carotene.^30^ The increased photoactivation yield observed with excitation above the 0–1 vibronic transition indicates that an excited-state barrier restricts the internal rotations with respect to the C=C bonds. As reviewed previously,^40^ the initial displacement in the S_2_ state from the Franck–Condon structure would be expected along the C=C and C–C stretching coordinates of the isoprenoid backbone, which would enhance the ability for out-of-plane twisting and bending motions tending toward a zwitterionic electronic configuration^48,76^ to occur during the longer lifetime of the S_*x*_ state. It is interesting to note that a degree of color sensitivity, favoring detection of blue incident photons preferentially over green photons, arises here from the underlying reaction dynamics of the OCP photoreceptor response. Similar excited-state conformational mechanisms are attributed to the cyanobacteriochromes, which also serve as photoreceptors in cyanobacteria.^77–80^ In diametrical contrast to the rhodopsins, where the quantum yield for the retinal photochemistry is enhanced compared to that observed in solution,^60^ the low effective quantum yield due to the protein’s dynamical response avoids a full depletion of the reservoir of resting OCP^O^ even at high light intensities. These conclusions raise the suggestion that the photoregulatory mechanism mediated by the OCP in cyanobacteria can be optimized to respond to changes in environmental conditions by mutations that tune the free-energy landscape of the folded protein and its interactions with the ketocarotenoid chromophore.

**Figure 9:**
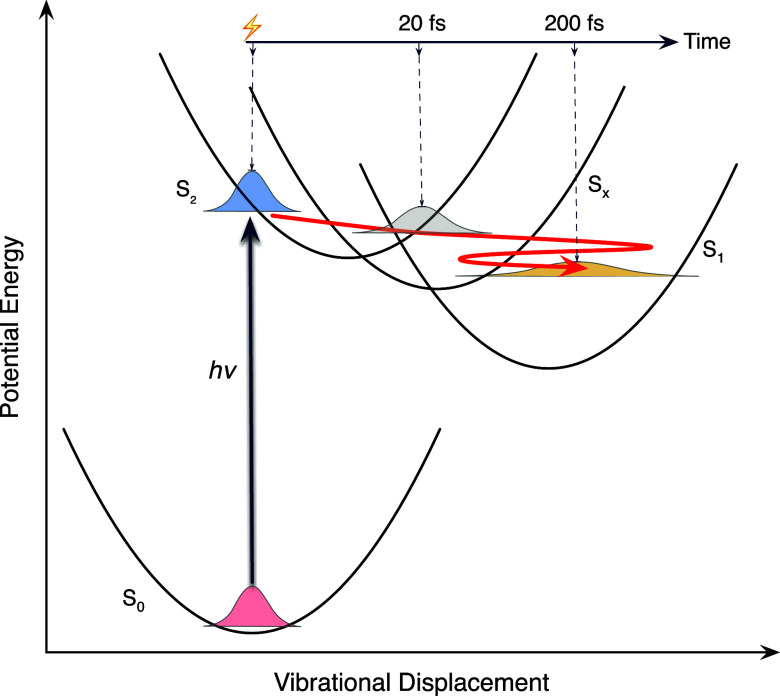
Coherent nonadiabatic mechanism for nonradiative relaxation from the optically prepared S_2_ state of canthaxanthin in the OCP. Approximate times for the arrival of the excited-state wavepacket at the S_*x*_ and S_1_ states are indicated for canthaxanthin in toluene solution, as measured previously in broadband multidimensional electronic spectroscopy experiments.^20^

## Supplementary Material



## Data Availability

Julia and Python code used to analyze the data reported in this paper will be provided in an archive at the following DOI: 10.5281/zenodo.14503092. There are no restrictions on the use of the code in the archive.

## Notes

Temperature dependence of the linear absorption spectrum of CAN in 2-MTHF over the 80–293-K range and tracking of the vibronic transitions; absorption, fluorescence, and fluorescence anisotropy spectra of CAN in 2-MTHF with excitation of the 0–0 vibronic transition; rotation of the emission transition dipole moment of β-carotene in 2-MTHF with respect to temperature; absorption, fluorescence, and fluorescence anisotropy spectra of zeaxanthin in 2-MTHF at 293 K; Arrhenius plot of the initial rate of photoactivation of OCP^O^; determination of the rate constants *k*_1_ and *k*_2_ for the photoactivation of OCP with 450 nm illumination; Gibbs free-energy surface for canthaxanthin in 2-MTHF solvent; table of hydrogen-bonding distances between the β1 carbonyl of CAN and the neighboring hydrogen-bond donors Y201 and W288 for the ground state and the *p* and *q* intermediates; box plot for the distribution of hydrogen-bond lengths to the Y201 and W288 residues from the WT-metaD simulations; and representative snapshot from the WT-metaD calculations showing the distance and angles of the hydrogen-bonding interactions with Y201 and W288 (PDF)
