# Supplementary material for: Photoactivation Transition State and Dynamical Response of the Orange Carotenoid Protein
Source: J Phys Chem B. 2025 Dec 4;129(50):12841–52. doi: 10.1021/acs.jpcb.5c05483 (PMC12720242; doi:10.1021/acs.jpcb.5c05483)
Supplement: Supplementary file 1 [file jp5c05483_si_001.pdf]

Supporting Information

Photoactivation transition state and dynamical response of the orange carotenoid protein

Justin B. Rose[1], José A. Gascón[2], Damien I. Sheppard[3],
Cheryl A. Kerfeld[3,4,5], and Warren F. Beck[1,*]

[1]Department of Chemistry, Michigan State University
578 S. Shaw Lane, East Lansing, Michigan 48824 U.S.A.

[2]Department of Chemistry, University of Connecticut
55 N. Eagleville Road, Storrs, Connecticut 48824 U.S.A.

[3]MSU–DOE Plant Research Laboratory, Michigan State University
612 Wilson Road, East Lansing, Michigan 48824 U.S.A.

[4]Environmental Genomics and Systems Biology Division
Lawrence Berkeley National Laboratory
1 Cyclotron Road, Berkeley, California 94720 U.S.A.

[5]Molecular Biophysics and Integrated Bioimaging Division
Lawrence Berkeley National Laboratory
1 Cyclotron Road, Berkeley, California 94720 U.S.A.

[*]Corresponding author. Email: beckw@msu.edu

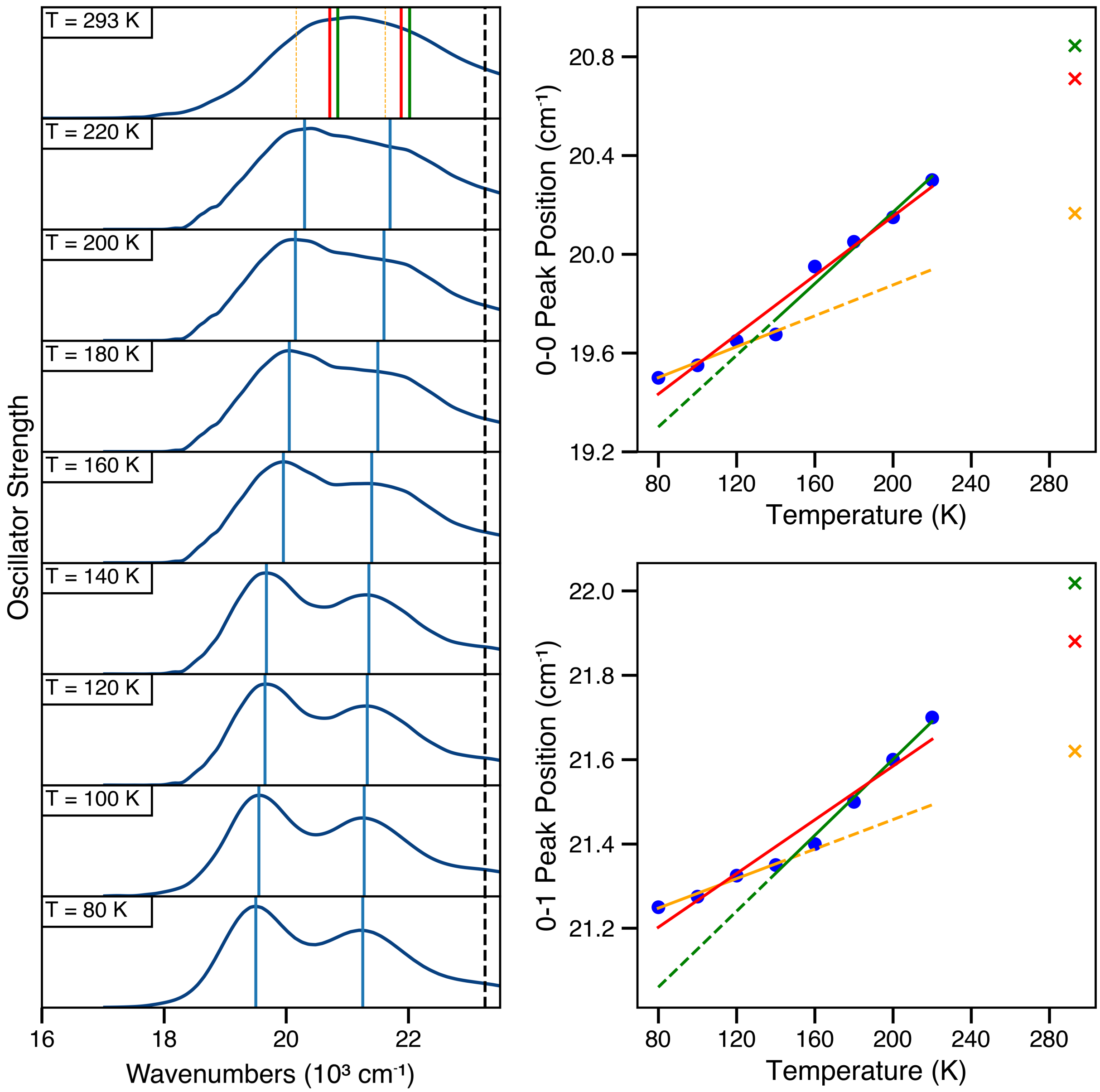

Fig. S1. (*left*) Temperature dependence of the linear absorption spectrum of canthaxanthin in 2-MTHF solvent over the 80–293-K range. The positions of the 0–0 and 0–1 vibronic transitions are marked for each spectrum by vertical lines; a dashed vertical line marks the 430-nm wavelength used in certain photoexcitation experiments. (*right*) The 0–0 and 0–1 peak positions are plotted as a function of temperature, with fitted lines for the low and high temperature linear trends. The crossing of the fitted segments coincides with the 142-K dynamical transition of the 2-MTHF solvent.

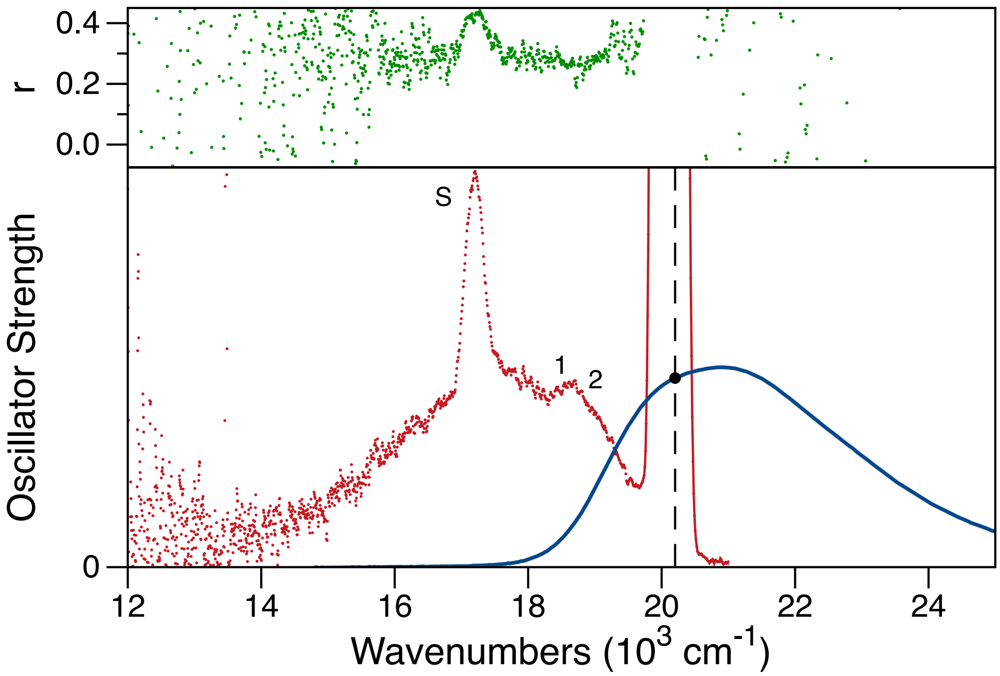

Fig. S2. Absorption (blue), fluorescence emission (red), and anisotropy r (green) spectra of canthaxanthin in 2-methyltetrahydrofuran (2-MTHF) solvent at 293 K, with excitation at the 0–0 vibronic transition, 495 nm (20200 cm^{-1}. The absorption and fluorescence spectra are plotted with arbitrary scaling as the oscillator strengths, $\varepsilon(\nu)/\nu$ and $\lambda^2 F(\nu)/\nu^3$, respectively. The fluorescence spectrum was recorded with the emission polarization at the magic angle, 54.7°, relative to the excitation polarization. The positions of resonance Raman scattering peaks of canthaxanthin at 1517 cm^{-1} and 1157 cm^{-1} are marked by the labels 1 and 2, respectively.[1] The non-resonant Raman scattering peak from the 2-MTHF solvent at 2966 cm^{-1} is marked by the label S.[2]

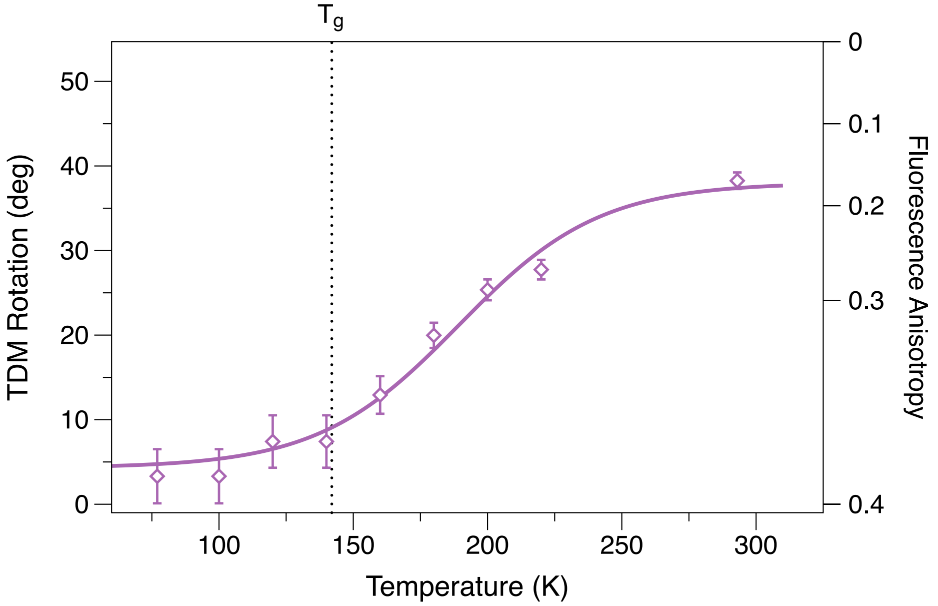

Fig. S3. Rotation of the emission transition dipole moment (TDM) of β-carotene in 2-MTHF solution, as determined from measurements of the fluorescence anisotropy, with excitation of the 0–2 absorption transition. The glass transition temperature for 2-MTHF, T_g = 142 K,[3] is marked by a vertical dotted line.

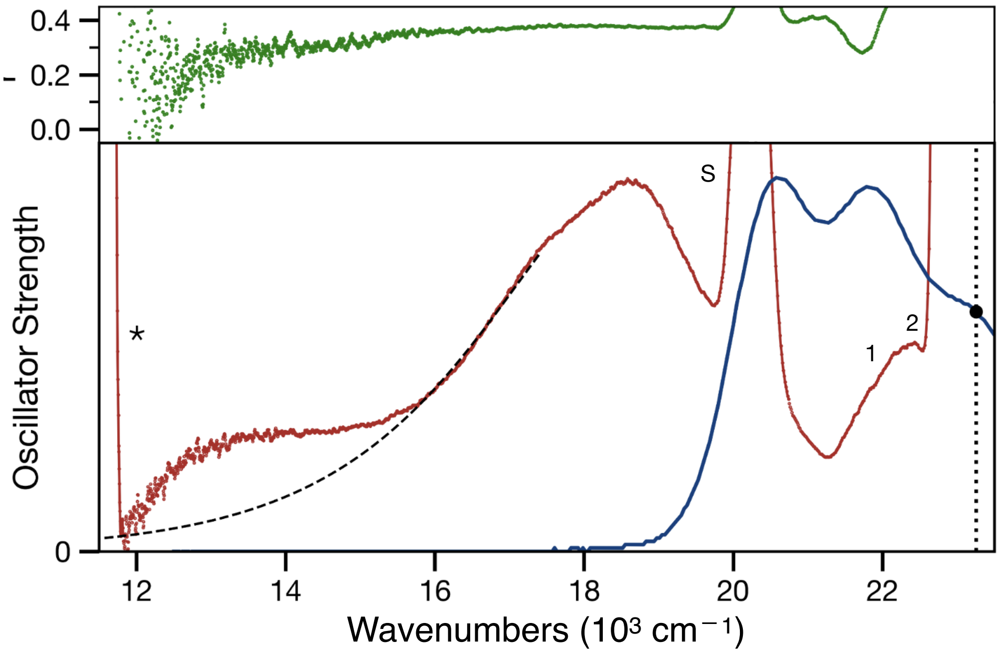

Fig. S4. Absorption (blue), fluorescence emission (red), and anisotropy r (green) spectra of zeaxanthin (ZEA) in 2-methyltetrahydrofuran (2-MTHF) solvent at 293 K. The absorption and fluorescence spectra are plotted with arbitrary scaling as the oscillator strengths, $\varepsilon(\nu)/\nu$ and $\lambda^2 F(\nu)/\nu^3$, respectively. The fluorescence spectrum was recorded with the emission polarization at the magic angle, 54.7°, relative to the excitation polarization. The excitation source was tuned to 430 nm, as marked by the vertical dashed line at 23250 cm^{-1}, which corresponds to excitation of the 0–2 vibronic transition. The positions of resonance Raman peaks of zeaxanthin[1] at 1517 cm^{-1} and 1157 cm^{-1} are marked by the labels 1 and 2, respectively. The non-resonant Raman scattering peak from the 2-MTHF solvent[2] at 2966 cm^{-1} is marked by the label S. Scattered light from the excitation source contributes a peak at 11625 cm^{-1} marked with an asterisk (*) due to the second order of the grating. The dotted curve extrapolates the main emission band into the near-IR region, which estimates the baseline for the emission band from S_1 near 13000 cm^{-1}.

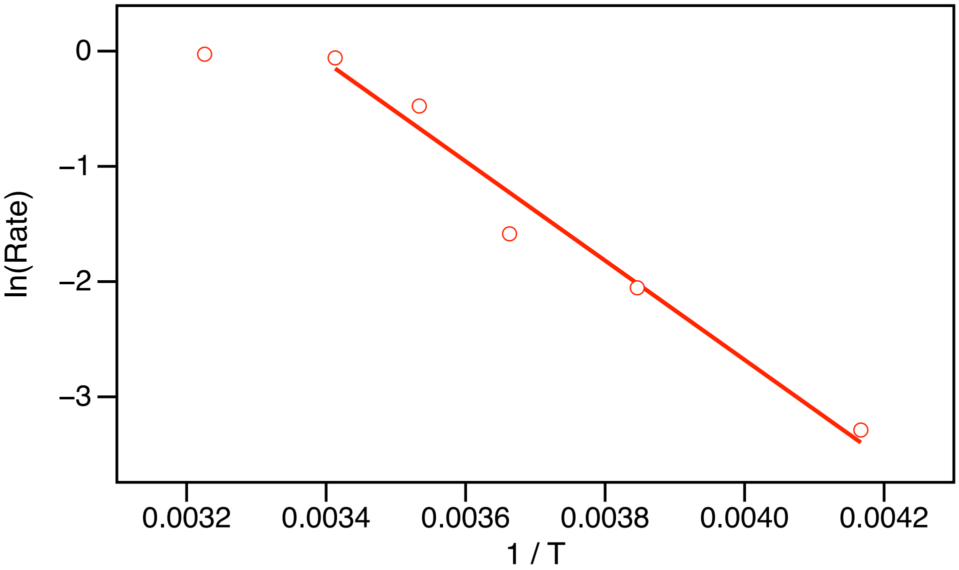

Fig. S5. Arrhenius plot of the initial rate of photoactivation of OCP. The data values plotted in Fig. 6 in the main manuscript are replotted here with respect to logarithmic rate and reciprocal temperature axes. The linear fit above returns an estimate of 8.5 kcal/mol for the activation energy E_a, with the highest temperature point excluded.

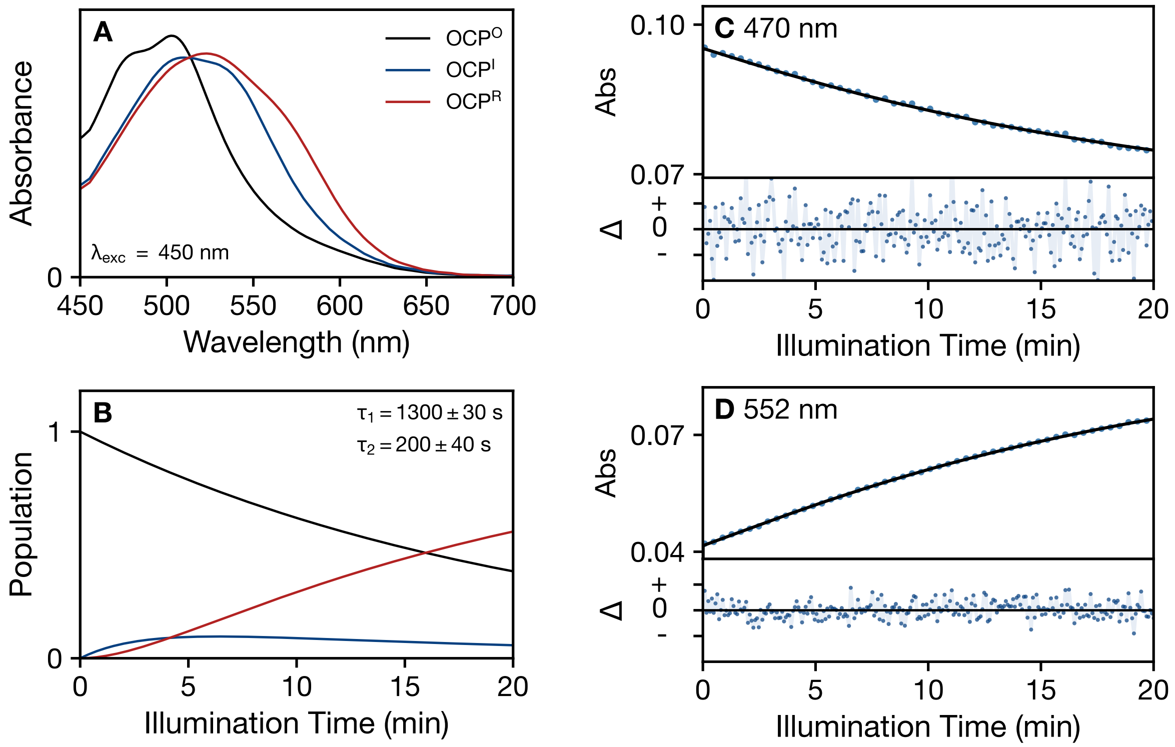

Fig. S6. Determination of the rate constants for the two steps in the photoactivation mechanism of OCP with 250 μW illumination at 450 nm using a global model with three spectrokinetic species, $OCP^O \xrightarrow{k_1} OCP^I \xrightarrow{k_2} OCP^R$, using the methods, conditions, and analysis discussed previously.[4] (A, B) Evolution-associated absorption spectra and time evolution of the populations for the three spectrokinetic species. The rate constants are evaluated as $k_1 = 1/\tau_1$ and $k_2 = 1/\tau_2$. (C, D) Fits with respect to illumination time of the global model (black curves) to the measured absorption values (blue dots) at two detection wavelengths, 470 nm and 552 nm. The residuals (Δ = observed − global model) are plotted with a 20× factor for the absorbance ordinate compared to that in the main panel.

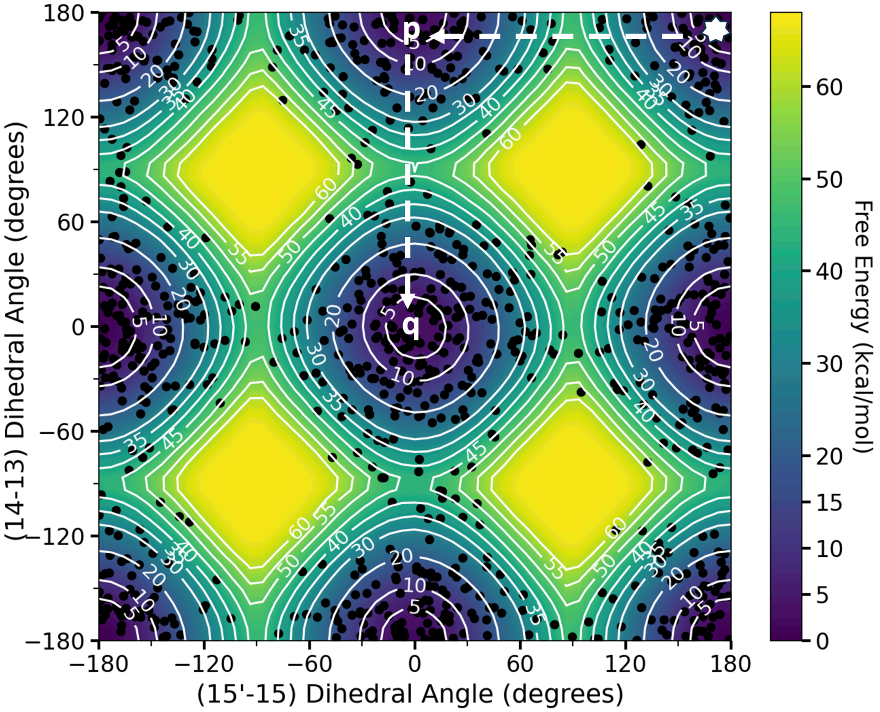

Fig. S7. Gibbs free-energy surface for canthaxanthin in 2-MTHF solvent, for comparison with that shown in the main manuscript in Fig. 8 for canthaxanthin in OCP^O. The marked coordinates indicate the all-*trans* energy minimum in the electronic ground state of canthaxanthin ($*$) and two adjacent local minima (p and q). To transition to p directly from the all-*trans* configuration, the barrier is ~45 kcal/mol, and the barrier from p to q is also ~45 kcal/mol.

Tab. S1. Distances[a] between the β1 carbonyl of canthaxanthin and the neighboring hydrogen bond donors Y201 and W288 for the ground state, as sampled in the WT-metaD simulations[5] in the ground state, p, and q minima of the free energy surface of OCP^O along the path to a bicycle-pedal configuration (Fig. 8).

Structure	Snapshots	Y201	W288
dark	44	2.33 ± 0.42 Å	1.85 ± 0.15 Å
p	7	3.56 ± 0.74 Å	2.31 ± 0.87 Å
q	26	2.56 ± 0.47 Å	1.96 ± 0.24 Å

a. The tabulated distances are for the hydrogen bonding vectors drawn in Fig. 8b in the main manuscript, and a snapshot from the WT-metaD simulations is shown here in Fig. S9. The average and standard deviations are determined over the indicated number of snapshots sampled in the three potential energy minima.

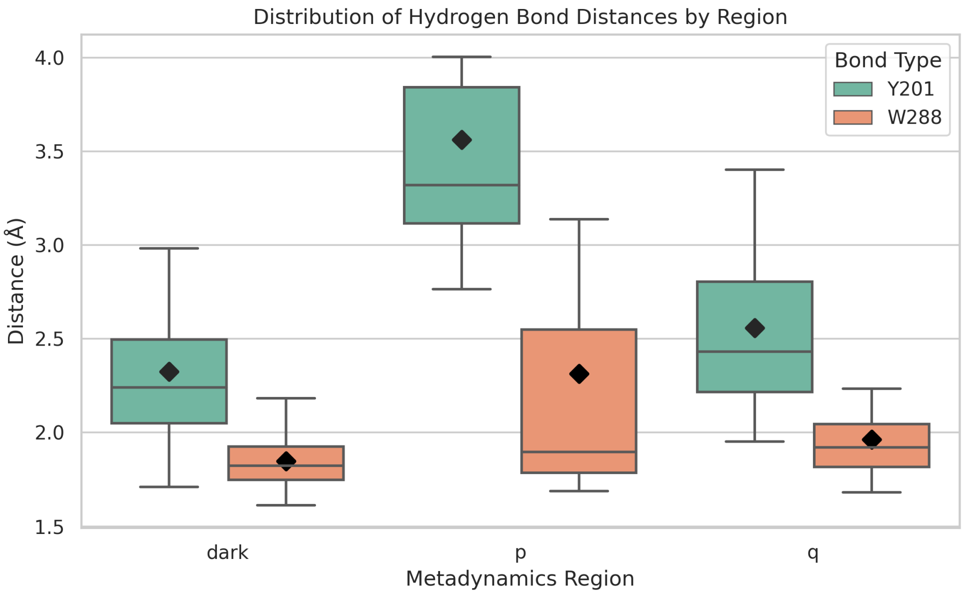

Fig. S8. Box plot for the distribution of hydrogen-bond lengths to the Y201 and W288 residues from the β1 carbonyl group from the WT-metaD simulations.[5]

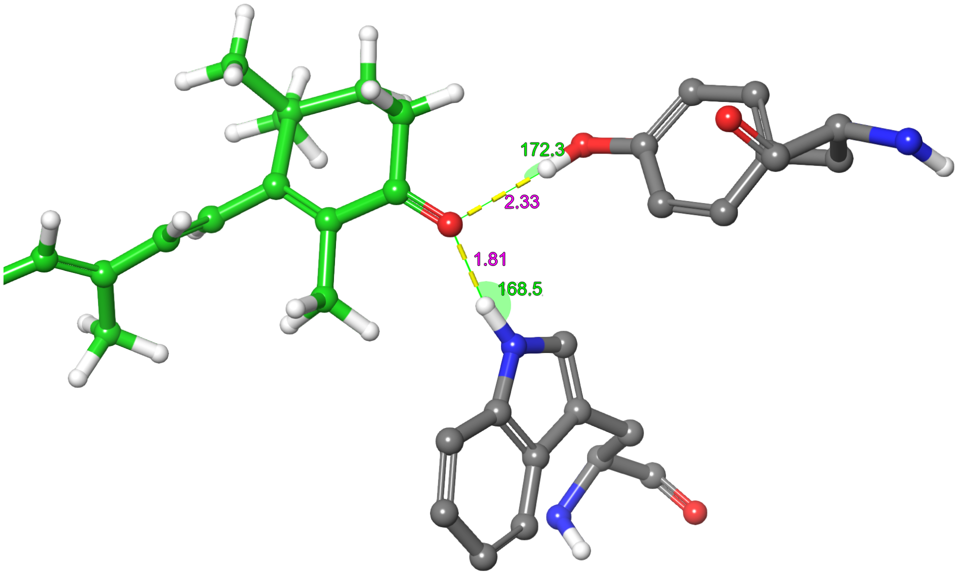

Fig. S9. A representative snapshot from the WT-metaD simulations in the dark ground-state of OCP showing the distances and angles for the hydrogen- bonding interactions with Y201 and W288.[5]

References

[1] Saito, S.; Tasumi, M.; Eugster, C. H. Resonance Raman spectra (5800–40 cm^{-1}) of all-*trans* and 15-*cis* isomers of β-carotene in the solid state and in solution. Measurements with various laser lines from ultraviolet to red. *J. Raman Spectrosc.* **1983**, *14*, 299–309, DOI: 10.1002/jrs.1250140503.

[2] Sverdlov, L. M.; Kovner, M. A.; Kraĭnov, E. P. *Vibrational Spectra of Polyatomic Molecules*; Wiley, 1973.

[3] Tan, R.-R.; Shen, X.; Hu, L.; Zhang, F.-S. Liquid-to-glass transition of tetrahydrofuran and 2-methyltetrahydrofuran. *Chin. Physics B* **2012**, *21*, 086402, DOI: 10.1088/1674-1056/21/8/086402.

[4] Rose, J. B.; Gascón, J. A.; Sutter, M.; Sheppard, D. I.; Kerfeld, C. A.; Beck, W. F. Photoactivation of the orange carotenoid protein requires two light-driven reactions mediated by a metastable monomeric intermediate. *Phys. Chem. Chem. Phys.* **2023**, *25*, 33000–33012, DOI: 10.1039/d3cp04484j.

[5] Barducci, A.; Bussi, G.; Parrinello, M. Well-tempered metadynamics: a smoothly converging and tunable free-energy method. *Phys. Rev. Lett.* **2008**, *100*, 020603, DOI: 10.1103/PhysRevLett.100.020603.
